# The Association Between Smartphone Addiction and Sleep: A UK Cross-Sectional Study of Young Adults

**DOI:** 10.3389/fpsyt.2021.629407

**Published:** 2021-03-02

**Authors:** Sei Yon Sohn, Lauren Krasnoff, Philippa Rees, Nicola J. Kalk, Ben Carter

**Affiliations:** ^1^Department of Biostatistics and Health Informatics, Institute of Psychiatry, Psychology and Neuroscience, King's College London, London, United Kingdom; ^2^Population Policy Practice, National Institute of Health Research, Great Ormond Street Hospital Biomedical Research Centre Institute of Child Health, University College London, London, United Kingdom; ^3^Addictions Department, Institute of Psychiatry, Psychology & Neuroscience, King's College London, London, United Kingdom; ^4^South London and Maudsley NHS Foundation Trust, Beckenham, United Kingdom

**Keywords:** smartphone addiction, sleep, smartphone harm reduction strategies, screen time, young adults, behavioral addiction

## Abstract

**Background:** In a large UK study we investigated the relationship between smartphone addiction and sleep quality in a young adult population.

**Methods:** We undertook a large UK cross-sectional observational study of 1,043 participants aged 18 to 30 between January 21st and February 30th 2019. Participants completed the Smartphone Addiction Scale Short Version, an adapted Pittsburgh Sleep Quality Score Index and reported smartphone use reduction strategies using both in-person (*n* = 968) and online (*n* = 75) questionnaires. A crude and adjusted logistic regression was fitted to assess risk factors for smartphone addiction, and the association between smartphone addiction and poor sleep.

**Results:** One thousand seventy one questionnaires were returned, of which 1,043 participants were included, with median age 21.1 [interquartile range (IQR) 19–22]. Seven hundred and sixty three (73.2%) were female, and 406 reported smartphone addiction (38.9%). A large proportion of participants disclosed poor sleep (61.6%), and in those with smartphone addiction, 68.7% had poor sleep quality, compared to 57.1% of those without. Smartphone addiction was associated with poor sleep (aOR = 1.41, 95%CI: 1.06–1.87, *p* = 0.018).

**Conclusions:** Using a validated instrument, 39% young adults reported smartphone addiction. Smartphone addiction was associated with poor sleep, independent of duration of usage, indicating that length of time should not be used as a proxy for harmful usage.

## Introduction

Smartphone use has become ubiquitous over the past decade. This has been accompanied by growing concerns around excessive and potentially harmful use (1). There are emerging reports of problematic behavior patterns in relation to smartphone use which mirror those of addiction (2). Although smartphone addiction is not formally recognized as a clinical diagnosis, it is a subject of active research. Validated instruments have been developed to characterize problematic smartphone use in terms of recognized dimensions of behavioral addiction with scores above which the research subject is considered to report smartphone addiction (3, 4). Research subjects reporting smartphone addiction describe a decreased interest in face-to-face relationships, use despite knowledge of the negative consequences, impaired control over and pre-occupation with their devices, and anxiety when their phones are inaccessible; these are not unlike the symptom domains of substance use disorders or other behavioral addictions (2, 5–7). Studies have highlighted associations of smartphone addiction with reduced productivity and with lower academic attainment (8, 9), demonstrating the negative functional impact on young people's lives and future prospects. Indirect harms resulting from smartphone addiction include propensity for accidents, for example through use whilst driving, and potential contribution to the obesity crisis by facilitating sedentary lifestyles (10, 11). Although at an early stage, there is also some neuroimaging evidence of volume and activity parallels between smartphone addiction and other addictions (12).

Studies to date have used the length of smartphone usage (measured as the total daily length of smartphone use) as an exposure indicative of problematic usage (13). However, while it is true that heavy use is seen in people with any addiction, it is also true that this is not sufficient for an addiction to be present, reflected in the ICD-11 criteria for gaming and gambling disorders (6) and in proposed diagnostic criteria for smartphone addiction (7). For an addiction to be present, subjective distress and functional impairment must also be present – in the case of smartphone addiction, neglect of other meaningful activities, pre-occupation with the phone, distress when access to the phone is not possible, and continued use despite evidence of harm. Measuring duration of use is an inexact proxy for addiction, as some people may experience the features of addiction with lower duration of use while others may use their phone in an adaptive way for long periods of time (for example, answering work emails during a long commute) but be able to put the phone down without distress and attend to appropriate activities such as communicating with family members, or going to bed on time (14).

It is important to note that smartphone addiction has several terms of reference including “nomophobia,” “problematic smartphone use,” and “smartphone dependence.” There is also a lack of consensus around whether putative “smartphone addiction” represents a distinct clinical identity and meets the criteria to be formally considered a behavioral addiction (15, 16). Furthermore, it remains unclear whether this dependence is on the smartphone itself or on the apps available through the device; whether the phone itself is like a substance of abuse or more like the needle through which addictive apps are delivered (14). There are similar patterns of behavior associated with specific applications (e.g., Facebook addiction, Instagram addiction) that are being investigated in their own right, and it is possible that certain types of phone use (e.g., social media use) may have more addictive implications than others (e.g., calling, texting), as the former involves display and expectation of approval through the creation, sharing, and viewing of content, while the latter replicates face-to-face relationships in terms of one-to-one communication (17, 18). Nevertheless, there is evidence of the existence of a behavioral phenotype that resembles addiction. The physical harms highlighted above, as well as emerging associations with psychiatric symptoms such anxiety and depressed mood indicate a pressing need to further investigate this growing phenomenon (19).

While the negative effects of screen time on sleep have been previously reported, smartphones are portable, hand-held devices that have much higher potential of interrupting sleep quality or quantity (20). Problematic smartphone use has been consistently linked to poor sleep in previous studies (4, 21), and smartphone overuse has been associated with daytime tiredness, longer sleep latency, and reduced sleep duration (22–24). In particular, smartphone use close to sleep initiation has been shown to delay circadian rhythm and found associated with total sleep time, where longer usage was associated with poor sleep (25). Furthermore, poor sleep outcomes may mediate the relationships between smartphone addiction and psychopathological symptoms (26). However, despite consistent advice from health bodies concerning the negative impacts of smartphone use on sleep, the majority of adults in the UK use their phones during the night and close to bed time (27).

A recent international systematic review found that the prevalence of smartphone addiction was around 25% in teenagers and young people (4). The weight of this evidence was from South and East Asia, and it has been noted that levels of smartphone addiction are often higher in Asian samples than in Western populations, possibly reflecting cultural practices around internet and smartphone use (28, 29). This study includes the largest UK sample to date to investigate the prevalence of smartphone addiction, and to clarify the association between smartphone addiction and sleep outcomes, in this population.

## Methods

### Study Design and Study Population

Participants were recruited opportunistically across multiple campuses at King's College London, England, between January 21st and February 4th, 2019. Participants were approached by researchers to describe the study, and invited to complete a paper-based case report form (CRF) based at four separate locations during the stated data collection period. Additionally, participants were invited to complete an identical online version of the CRF through an internal research recruitment process. Eligibility criteria included students at King's College London aged between 18 and 30 who owned a smartphone. Participants were excluded if they did not adequately complete the Smartphone Addiction Scale – Short version [SAS-SV [3]] or the adapted Pittsburgh Sleep Quality Index [PSQI (30)]. The study was undertaken in accordance with the Declaration of Helsinki. Ethical approval was received from the King's College Research Ethics Office (Study ID: 9138; MRS-18/19-9138) and the full protocol is available on request. All face to face participants provided informed verbal consent prior to involvement and those submitting online gave consent by responding to the questionnaire.

### Measures

The case report form (CRF) was co-developed amongst researchers, teenagers and young people with experience of smartphone use (Supplementary Table 1). The CRF included demographic information, smartphone use characteristics, a validated scale for smartphone addiction [SAS-SV (3)], an adapted sleep score based on the Pittsburgh Sleep Quality Instrument [PSQI (30)], and a range of reduction strategies. To reduce perception bias, the CRF included neutral non-directive phrasing about smartphone use collecting both positive and negative aspects.

#### Smartphone Use Characteristics

Participants were asked about the quantity of smartphone use (the average length of daily time) and the timing of use.

#### Smartphone Addiction Scale – Short Version (SAS-SV)

The SAS-SV is a 10-question validated scale that was developed to assess smartphone addiction in children (mean age of 14.5) (3). Participants are asked to rate statements related to their smartphone use, such as “Using smartphone longer than intended” on a 6-point Likert scale, from “strongly disagree” (1) to “strongly agree” (6). The resulting total score is between 10 and 60, with higher totals indicating higher risk of smartphone addiction. Total scores of 31 and 33 were used as diagnostic thresholds for males and females respectively, in accordance with the original study which found strong internal consistency (Cronbach's alpha = 0.91, AUC = 0.96 for boys, AUC = 0.89 for girls). This scale has been widely used internationally and has been found to have similarly strong internal consistencies using the same thresholds for this study's age group (31, 32).

#### Sleep

Participants were asked to rate their subjective sleep quality on an average weeknight on a Likert scale of 1–10 and the number of hours they slept on an average weeknight on a Likert scale of <4 to 12, taking into account the expected average number of hours of sleep for adults, in order to assess sleep quality and duration. Participants were additionally asked the number of days a week they felt noticeably tired or fatigued during the day (0–7) and the number of nights a week where they felt it difficult to fall asleep (0–7) to measure daytime tiredness and sleep latency. Based on these responses, scores for each component were calculated, which were then combined to calculate a global sleep score, adapted from the Pittsburgh Sleep Quality Index [PSQI (30)], where a score of ≤ 5 was considered good sleep (33).

#### Reduction Strategies

Commonly used strategies to reduce smartphone use included within the CRF were identified from the literature and through consultation with subject matter experts and young people (Supplementary Table 2). Participants were asked to rate the effectiveness of any strategies employed from ineffective to very effective.

### Sample Size Justification

Estimates of the prevalence of poor sleep prevalence are wide-ranging. At study conception it was estimated that 42% of participants without problematic smartphone usage would exhibit poor sleep (34), and this would increase to 55% in those exhibiting problematic smartphone usage (4). In order to detect this difference using an independent chi-squared test of proportions with 90% power and 5% significance, we would need to include 650 participants. Building on this, to account for 15% missing data, we would need to include at least 780 participants in total.

### Outcomes

The primary outcome was the association between sleep quality and smartphone addiction. Secondary outcomes were to determine: the association of smartphone addiction with demographics and usage characteristics; and the impact of reduction strategies on mitigating the effect of smartphone addiction on sleep.

### Statistical Analysis

Demographic and smartphone usage characteristics of the sample were summarized, comparing participants with good, and poor sleep. Crude logistic regression models were included, to assess poor sleep and demographic (site, sex, and age) and smartphone usage characteristics. An adjusted multivariable logistic regression was carried out fitting the demographic with important characteristics found from the crude univariable analyses.

Both crude odds ratio[s] (OR) and adjusted OR (aOR) were presented alongside their respective 95% confidence intervals (95%CI), and *P*-values (<0.05 considered statistically significant). SPSS Versions 25 and 26 (IBM Corp., Armonk, N.Y., USA) were used to input and analyse data.

To determine the association between smartphone addiction and the demographic and usage characteristics, a multivariable logistic model adjusting for the same covariates as for the primary outcome was created. Due to multi co-linearity total usage, latest time of use and usage cessation prior to sleep were not fitted within the same analysis models.

#### Missing Data and Population Under Investigation

Individuals with missing item data of no more than 30% of the SAS-SV, or the adapted sleep score were proportionally mean imputed (35). Due to the completeness of the data collected, a complete case analysis was used.

#### Role of the Funding Source

There was no funding source for this study. The corresponding author had full access to all the data in the study and had final responsibility for the decision to submit for publication.

## Results

### Characteristics of the Participants

We received 1,071 completed CRFs, of which 1,043 participants were eligible and included (completion rate 97.8%). The 28 excluded participants were ineligible due to age, or non-completion of the SAS-SV or items from the PSQI score. Of those included, 38 of the SAS-SV score, and 85 of the adapted PSQI had a single item of each domain missing and were (within participant) domain mean-imputed.

The mean age of the included participants was 21.1 (IQR 19–22, range 18–30), where 92.1% (*n* = 961) were aged under 26, and 73.2% (*n* = 763) of the participants were female (Table 1). In terms of smartphone usage, 23.7% (*n* = 247) used their phones for 3 h per day, while 18.5% (*n* = 193) used their phones for more than 5 h daily. A large proportion of the young adult population exhibited poor sleep quality (61.6%, *n* = 643). Of those exhibiting smartphone addiction, 68.7% (*n* = 279) had poor sleep quality compared to 57.1% (*n* = 364) of those not exhibiting smartphone addiction. Of those participants that ceased smartphone use with 30 min of initiating sleep, 61.4% (*n* = 478) had poor sleep, compared to 53.6% (*n* = 45) of those who ceased use more than 1 h prior to initiating sleep.

**Table 1:** The included population sociodemographic and phone use characteristics.

**Included participant characteristics**	**Full sample**		**Good sleep**		**Poor sleep**	
	***n***	**%**	***n***	**%**	***n***	**%**
	1,043		400	38.4	643	61.6
**Age**						
≤ 21	680	65.2	247	36.3	433	63.7
22-25	281	26.9	116	41.3	165	58.7
≥26	82	7.9	37	45.1	45	54.9
**Gender**						
Male	280	26.8	115	41.1	165	58.9
Female	763	73.2	285	37.4	478	62.6
**Ethnicity**						
Asian	389	37.3	145	37.3	244	62.7
Black	86	8.2	28	32.6	58	67.4
White	452	43.3	186	41.2	266	58.8
Mixed	55	5.3	16	29.1	39	70.9
Other	16	1.5	6	37.5	10	62.5
Missing	45	4.3	19	42.2	26	57.8
**Year of degree**						
1st	387	37.1	135	34.9	252	65.1
2nd	169	16.2	65	38.5	104	61.5
3rd	212	20.3	81	38.2	131	61.8
4th	48	4.6	21	43.8	27	56.3
5th	42	4.0	15	35.7	27	64.3
Post-graduate (MSc, PhD, post-graduate certificates)	164	15.7	74	45.1	90	54.9
Missing	21	2.0	9	40.9	13	59.1
**Faculty**						
Arts and Humanities	67	6.4	24	35.8	43	64.2
Dentistry, oral, and craniofacial sciences	53	5.1	15	28.3	38	71.7
Nursing, midwifery, and palliative care	103	9.9	32	31.1	71	68.9
Business School	12	1.2	2	16.7	10	83.3
Law	61	5.8	22	36.1	39	63.9
Life Sciences and Medicine	383	36.7	149	38.9	234	61.1
Natural and Mathematical Sciences	73	7.0	32	43.8	41	56.2
Psychiatry, psychology, and neuroscience	154	14.8	70	45.5	84	54.5
Social Science and Policy	92	8.8	37	40.2	55	59.8
Missing	45	4.3	17	37.8	28	62.2
**Recruitment location (*****p*** **=** 0.007)*						
1	334	32.0	129	38.6	205	61.4
2	335	32.1	111	33.1	224	66.9
3	144	13.8	69	47.9	75	52.1
4	155	14.9	68	43.9	87	56.1
5	75	7.2	23	30.7	52	69.3
**Smartphone addiction (*****p*** **<** **0.001)**						
Not addicted	637	61.1	273	42.9	364	57.1
Addicted	406	38.9	127	31.3	279	68.7
**Total daily hours (*****p*** **=** **0.004)**						
≤ 2 h	207	19.8	103	49.8	104	50.2
3 h	247	23.7	91	36.8	156	63.2
4 h	226	21.7	77	34.1	149	65.9
5 h	162	15.5	65	40.1	97	59.9
>5 h	193	18.5	62	32.1	131	67.9
Missing	8	0.8	2	25.0	6	75.0
**Latest time of use (*****p*** **<** **0.001)**						
Before 11 p.m.	122	11.7	70	57.4	52	42.6
11:00	102	9.8	32	31.4	70	68.6
11:30	134	12.8	72	53.7	62	46.3
12:00	168	16.1	65	38.7	103	61.3
12:30	136	13.0	54	39.7	82	60.3
1 a.m. or later	353	33.8	102	28.9	251	71.1
Missing	28	2.7	5	17.9	23	82.1
**Smartphone cessation prior to sleep initiation**						
<30 min	778	74.6	300	38.6	478	61.4
30 min−1 h	151	14.5	55	36.4	96	63.6
>1 h	84	8.1	39	46.4	45	53.6
Missing	30	2.9	6	20.0	24	80.0

* Recruitment location.

1, Life Sciences & Medicine campus.

2, Life Sciences, Nursing & Midwifery, and Social Sciences campus.

3, Psychiatry, psychology, and neuroscience campus.

4, Arts & Humanities, Law, Business, Natural & Mathematical Sciences, and Social Sciences & Public Policy campus.

*5, Online*.

### Prevalence of Smartphone Addiction, Sociodemographic Characteristics and Smartphone Usage

The overall prevalence of smartphone addiction was 38.9% (95%CI: 35.9–41.9%; *n* = 406**/**1,043). This includes 35.7% of males who were addicted and 40.1% of females (**Table 3**). For participants aged under 21 years, 42.2% exhibited smartphone addiction, compared to 34.2 and 28.0% of participants aged 22–25 years, and over 26 years, respectively. Of participants who used their smartphone for 2 or less hours per day, 20.3% were addicted, compared to 53.9% of those who used it for more than 5 h. Of those that stopped using their device more than an hour before bedtime, 23.8% exhibited addiction, compared to 42.0% of those stopping <30 min before bedtime (**Table 3**).

### Primary Outcome of Poor Sleep Associated With Smartphone Usage

We assessed demographic factors', phone usage characteristics', and reduction strategies' associations with poor sleep. Age, sex or site were not associated with poor sleep (Table 2). There was an association between poor sleep and those addicted (compared to not addicted, OR = 1.65, 95%CI:1.27–2.14, *p* < 0.001); and screen time (compared to 3, 2 h OR = 0.59, 95%CI 0.41–0.86, *p* = 0.007).

**Table 2:** The association between poor sleep and smartphone addiction, using crude and multivariable logistic regression.

**Variable**	**Total: *n* (%)**	**Crude OR (95%CI)**	***p*-value**	**Adjusted OR^**†**^ (95% CI) (*n* = 995)‡**	***p*-value**
**Age**					
≤ 21	680 (65.2)	1.00	-	1.00	-
22–25	281 (26.9)	0.81 (0.61–1.08)	0.150	0.95 (0.69–1.32)	0.767
≥2	82 (7.9)	0.69 (0.44–1.10)	0.121	0.85 (0.51–1.42)	0.540
**Gender**					
Male	280 (26.8)	1.00	-	1.00	-
Female	763 (73.2)	1.17 (0.88–1.55)	0.274	1.08 (0.79–1.46)	0.637
**Recruitment Site§**					
1	334 (32.0)	1.00	-	1.00	-
2	336 (32.1)	1.27 (0.93–1.74)	0.139	1.13 (0.80–1.58)	0.498
3	144 (13.8)	0.68 (0.46–1.02)	0.059	0.69 (0.45–1.08)	0.102
4	155 (14.9)	0.81 (0.55–1.19)	0.271	0.83 (0.55–1.25)	0.369
5	75 (7.2)	1.42 (0.83–2.44)	0.199	1.37 (0.77–2.42)	0.283
**SAS-SV**					
Not addicted	637 (61.1)	1.00	-	1.00	-
Addicted	406 (38.9)	1.65 (1.27–2.14)	<0.001**	1.41 (1.06–1.87)	0.018*
**Total daily hours**					
≤ 2	207 (19.8)	0.59 (0.41–0.86)	0.006**	0.62 (0.42–0.92)	0.018*
3	247 (23.7)	1.00	-	1.00	-
4	226 (21.7)	1.13 (0.77–1.65)	0.529	1.01 (0.68–1.49)	0.957
5	162 (15.5)	0.87 (0.58–1.31)	0.504	0.73 (0.47–1.12)	0.152
>5	193 (18.5)	1.23 (0.83–1.83)	0.303	1.10 (0.72–1.69)	0.653
**Smartphone at night**					
In bedroom	625 (59.9)	1.00	-	1.00	-
In other room	409 (39.2)	0.95 (0.74–1.23)	0.718	1.02 (0.77–1.34)	0.913
Missing	9 (0.9)				

Crude Odds Ratio (OR) and adjusted ORs. OR, odds ratio; CI, confidence interval; SAS-SV, Smartphone Addiction Scale–Short Version.

* p <0.05;

** p <0.01.

Adjusted for age, gender, ethnicity, recruitment site, smartphone addiction, total daily hours and smartphone location at night. Model fit with the adjusted Chi-squared = 36.760, p = 0.002.

‡ 52 participants were excluded due to having missing covariate data.

§ Recruitment location.

1, Life Sciences & Medicine campus.

2, Life Sciences, Nursing & Midwifery, and Social Sciences campus.

3, Psychiatry, psychology, and neuroscience campus.

4, Arts & Humanities, Law, Business, Natural & Mathematical Sciences, and Social Sciences & Public Policy campus.

*5, Online*.

In the multivariable analysis after adjustment for age, gender, site, screen time, and location of phone at night, those addicted exhibited a 41% increased odds of poor sleep (aOR = 1.41, 95%CI: 1.06–1.87, *p* = 0.018) (Table 2). Age, sex or site was not significantly associated with poor sleep. Total daily use of 2 or less hours reduced odds of poor sleep by 38% (aOR = 0.62, 95%CI: 0.42–0.92, *p* = 0.018).

### Secondary Outcome of Demographics Associated With Smartphone Addiction

In a crude analysis, age, site and ethnicity were associated with smartphone addiction (Table 3). There was a decreased odds of smartphone addiction in older groups, with 22–25 year olds having a 29% decreased odds compared with those 21 and younger (OR = 0.71, 95%CI: 0.53–0.95, *p* = 0.015), and participants 26 or older having a 47% decreased odds (OR = 0.53, 95%CI:0.32–0.89, *p* = 0.015). Those of Asian ethnicity had increased odds of addiction (OR = 1.75, 95%CI: 1.32–2.32, *p* < 0.001) when compared to a White reference population.

**Table 3:** The association between sociodemographic factors and smartphone addiction, using crude and multivariable logistic regression.

**Variable**	**Not Addicted *n* (%)**	**Smartphone Addicted *n* (%)**	**Crude OR (95%CI)**	***p*-value**	**Adjusted OR**** (95%CI)**	***p*-value**
**Age**						
≤ 21	393 (57.8)	287 (42.2)	1.00		1.00	
22-25	185 (65.8)	96 (34.2)	0.71 (0.53–0.95)	0.021	0.77 (0.55–1.08)	0.129
≥26	59 (72.0)	23 (28.0)	0.53 (0.32–0.89)	0.015	0.55 (0.31–0.96)	0.036*
**Gender**						
Male	180 (64.3)	100 (35.7)	1.00	-	1.00	-
Female	457 (59.9)	306 (40.1)	1.21 (0.91–1.60)	0.198	1.06 (0.78–1.45)	0.711
**Ethnicity**						
Asian	212 (54.5)	177 (45.5)	1.75 (1.32–2.32)	<0.001	1.49 (1.10–2.01)	0.010*
Black	50 (58.1)	36 (41.9)	1.51 (0.94–2.42)	0.087	1.15 (0.69–1.90)	0.598
White	306 (67.7)	146 (32.3)	Ref.	-	1.00	-
Mixed & other	44 (62.0)	27 (38.0)	1.29 (0.77–2.16)	0.341	1.18 (0.69–2.03)	0.542
Missing						
**Recruitment site**‡						
1	195 (58.4)	139 (41.6)	1.00	-	1.00	-
2	191 (57.0)	144 (43.0)	1.06 (0.78–1.44)	0.720	0.81 (0.58–1.13)	0.218
3	99 (68.8)	45 (31.3)	0.64 (0.42–0.97)	0.033*	0.74 (0.47–1.17)	0.201
4	108 (69.7)	47 (30.3)	0.61 (0.41–0.92)	0.017*	0.61 (0.40–0.95)	0.027*
5	44 (58.7)	31 (41.3)	0.99 (0.60–1.64)	0.964	0.99 (0.57–1.71)	0.966
**Daily total hours**						
≤ 2	165 (79.7)	42 (20.3)	0.55 (0.36 - 0.85)	0.007**	0.61 (0.39–0.96)	0.031*
3	169 (68.4)	78 (31.6)	1.00		1.00.	-
4	123 (54.4)	103 (45.6)	1.81 (1.25–2.64)	0.002**	1.75 (1.19–2.57)	0.005**
5	88 (54.3)	74 (45.7)	1.82 (1.21–2.74)	0.004**	1.67 (1.09–2.57)	0.019*
>5	89 (46.1)	104 (53.9)	2.53 (1.71–3.74)	<0.001**	2.45 (1.63–3.69)	<0.001**

Crude Odds Ratio (OR) and adjusted ORs. OR, odds ratio; CI, confidence interval; SAS-SV, Smartphone Addiction Scale–Short Version.

* p <0.05;

** p <0.01.

Adjusted for age, gender, ethnicity, recruitment site and hours total. Model fit with the adjusted Chi-squared = 77.801, p <0.001.

‡ Recruitment location.

1, Life Sciences & Medicine campus.

2, Life Sciences, Nursing & Midwifery, and Social Sciences campus.

3, Psychiatry, psychology, and neuroscience campus.

4, Arts and Humanities, Law, Business, Natural & Mathematical Sciences, and Social Sciences & Public Policy campus.

*5, Online*.

The total number of hours spent on smartphones per day was significantly and positively associated with the SAS-SV score. After adjustment, age, ethnicity, site and screen time were associated with addiction.

### Secondary Outcome of Smartphone Usage Characteristics and Addiction

Use for 2 h or less per day showed significantly decreased odds of smartphone addiction, compared with a reference of 3 h (OR = 0.55, 95%CI 0.36–0.85, *p* = 0.007, Table 4). Use for 5 or more hours per day showed a 2.5 times increase in odds (OR = 2.53, 95%CI: 1.71–3.74, *p* < 0.001). After adjustment for confounding factors, a consistent pattern of association was found between usage characteristics and addiction. There was a 39% reduction in odds of addiction for those using their phones for 2 h or less compared with typical usage of 3 h (aOR = 0.61; 95%CI 0.39–0.96; *p* = 0.031).

**Table 4:** The association between smartphone use characteristics and smartphone addiction, using crude and multivariable logistic regression.

**Variables**	**Not addicted *n* (%)**	**Smartphone addicted *n* (%)**	**Crude OR (95%CI)**	***p*-value**	**Adjusted OR**** (95%CI)**	***p*-value**
**Total hours spent on phone per day**						
≤ 2	165 (79.7)	42 (20.3)	0.55 (0.36–0.85)	0.007**	0.61 (0.39–0.96)	0.031*
3	169 (68.4)	78 (31.6)	Ref.	-	Ref.	-
4	123 (54.4)	103 (45.6)	1.81 (1.25–2.64)	0.002**	1.75 (1.19–2.57)	0.005**
5	88 (54.3)	74 (45.7)	1.82 (1.21–2.74)	0.004**	1.67 (1.09–2.57)	0.019*
>5	89 (46.1)	104 (53.9)	2.53 (1.71–3.74)	<0.001**	2.45 (1.63–3.69)	<0.001**
**Latest time on phone**						
Before 11 p.m.	98 (80.3)	24 (19.7)	Ref.	-	Ref.	-
11:00	78 (76.5)	24 (23.5)	1.26 (0.66–2.38)	0.484	1.14 (0.58–2.24)	0.697
11:30	92 (68.7)	42 (31.3)	1.86 (1.05–3.32)	0.034*	1.82 (1.00–3.32)	0.049*
12:00	99 (58.9)	69 (41.1)	2.85 (1.66–4.89)	<0.001**	2.51 (1.42–4.43)	0.002**
12:30	74 (54.4)	62 (45.6)	3.42 (1.96–5.99)	<0.001**	3.29 (1.83–5.90)	<0.001**
1 a.m. or later	177 (50.1)	176 (49.9)	4.06 (2.48–6.65)	<0.001**	3.91 (2.32–6.61)	<0.001**
**Time between latest phone use and bed time**						
1 h or more	64 (76.2)	20 (23.8)	Ref.	-	Ref.	-
30 min−1 h	103 (68.2)	48 (31.8)	1.49 (0.81–2.74)	0.198	1.47 (0.78–2.76)	0.230
<30 min	451 (58.0)	327 (42.0)	2.32 (1.38–3.91)	0.002**	2.17 (1.27–3.70)	0.004**

Crude Odds Ratio (OR) and adjusted ORs. OR, odds ratio; CI, confidence interval; SAS-SV, Smartphone Addiction Scale–Short Version.

* p <0.05;

** p <0.01.

*Adjusted for age, gender, ethnicity, and recruitment site. For total hours per day, model fit with the adjusted Chi-squared = 77.801, p <0.001. For latest time on phone, model fit with the adjusted Chi-squared = 78.502, p <0.001. For time between latest phone use and bed time, model fit with the adjusted Chi-squared, 43.977, p <0.001*.

Later time of usage was also significantly associated with smartphone addiction in a crude logistic regression analysis (Table 3). Use at 1 a.m. or later resulted in a four times increased risk of smartphone addiction, compared to those whose latest time of phone use was before 11 p.m. (OR = 4.06, 95%CI:2.48–6.65, *p* < 0.001). After adjustment, this finding remained consistent (aOR = 3.91, 95%CI:2.32–6.61, *p* < 0.001). Use within 30 min of initiating sleep resulted in a two times increased risk of smartphone addiction, which remained significant and consistent after adjustment (aOR = 2.17, 95%CI:1.27–3.70, *p* = 0.004).

### Smartphone Usage Reduction Strategies

92.1% of participants attempted at least one reduction strategy (Supplementary Tables 2, 3). The most popular strategies were putting your phone on “do not disturb” or in “airplane mode” at night (67.7%); turning off notifications (68.4%); and putting your phone on silent (85.1%). Those who reported smartphone addiction used more strategies than those who did not (mean difference = 0.28, 95%CI: 0.021–0.54, *p* = 0.034).

## Discussion

This study included 1,043 young adults at a UK university and examined the phenomenon of smartphone addiction. The prevalence of smartphone addiction was 38.9%. Smartphone addiction had associations with both ethnicity and age. Smartphone addiction was associated with poorer sleep.

Our estimated prevalence is consistent with other reported studies in young adult populations globally, which are in the range of 30–45%, and with Yang et al. (29) who studied a similar university population in the UK (4, 36–39). Noe et al. (40). estimate a UK prevalence of 19% using the SAS-SV with the same thresholds; however this study included an older population (up to 46 years) with a smaller sample size (*n* = 64). The inverse association between age and smartphone addiction highlighted in our study may explain this variation in prevalence estimates. It is likely that differences in prevalence across the field may be due to the varying criteria of instruments used, or different applications of cut-off scores, and we have previously outlined the differences between the most widely used instruments [Sohn et al. (4)].

Smartphone addiction was more prevalent amongst younger participants. This may reflect increased willingness amongst younger generations to adopt newer uses for smartphones (e.g., gaming, social media), which may confer greater risk of addiction (41). This could also related to younger participants potentially having more time for such endeavors. Participants from Asian ethnic backgrounds were at greater risk for smartphone indication, which may be due to cultural differences, such as social norms and characteristics including individualism (29, 42). There was no association between smartphone addiction and gender, at odds with other studies which have found that females are more at risk, but it should be noted that the SAS-SV applied a gender-based standardized threshold to determine addiction (43).

Longer use was significantly associated with smartphone addiction, which is consistent with other studies that have found that increased exposure is linked with increased dependency (44). Furthermore, later time of use was also significantly associated with smartphone addiction, with use after 1 a.m. conferring a 3-fold increased risk. This association may be indicative of impaired control and use despite harm, which are a characteristic of a behavioral addiction. Smartphone ownership has previously been linked with more electronic media use in the night and later bedtimes in a survey of adolescents (45).

Our study provides further support to the growing body evidence that smartphone addiction has a deleterious impact on sleep (16, 20, 23). However, this relationship remained significant after adjusting for daily screen time (which was not seen as predictive after adjustment for smartphone addiction). This finding suggests that although duration of exposure, as with any addiction, is a risk factor for smartphone addiction, it is not the only determining component, reflecting the ICD-11 criteria for gaming and gambling disorders, in which duration of use may be one component of diagnosis but is not the only indicator (6, 14). Furthermore, this result indicates that the relationship between sleep quality and smartphone addiction is not simply due to the duration of exposure, as suggested by other studies (46). It highlights that studies reporting a lack of association between smartphones and clinical outcome when using screen time alone should be interpreted with caution, as they have perhaps overlooked smartphone addiction as the harmful exposure (47).

The results of this study indicate that self-reported smartphone addiction is prevalent amongst young adults attending university and that it is linked with use at later times of the day in addition to total duration of use. Public health bodies should take this evidence into account when developing guidelines around smartphone use and sleep hygiene. Furthermore, clinicians, parents, and educators should be aware of the pervasiveness of smartphone addiction, and be prepared to consider the potential wide-reaching impact of smartphones on sleep. Despite the cross-sectional nature of this study, the findings suggest that the amount of time spent on their phones, and latest time of use can be indicative of those at risk for an addicted pattern of smartphone use. Should smartphone addiction become firmly established as a focus of clinical concern, those using their phones after midnight or using their phones for 4 or more hours per day are likely to be at high risk, and should guide administration of the SAS-SV. However, it should be noted that duration of smartphone use alone does not indicate smartphone addiction; it is merely indicates increased risk for development of this pattern of behavior. Future studies should examine longitudinal associations between smartphone use patterns and smartphone addiction, and between smartphone addiction and health harms, as well as exploring strategies to reduce harms, particularly in relation to sleep. As there is continued debate concerning the possibility that smartphones may be a means to access addictive material, such as social media applications or games, rather than the addiction themselves, future research should also focus on identifying types of use associated with higher risk of smartphone addiction.

This study collected data from a large sample of 18–30 year olds in the United Kingdom using a validated and widely used scale. There were several limitations to this study. Namely, due to the cross-sectional nature of data collection, no causal relationships can be drawn, and we cannot ignore the possibility of reverse causality. In particular, it is possible that poor sleep may be a result of concurrent mental health disorders that were not assessed for in this study, which may result in or be independently associated with increased smartphone usage and smartphone addiction risk. In addition, the self-reported data collection method we used may introduce common-method and response biases. Caution should be taken over the estimate of prevalence since a convenience sampling method was used. Additionally, caution should be taken in generalizing the results of this study, as the sampled population is not representative of the UK-wide population of young adults. Finally, these data were collected before the global pandemic, which may have led to a shift in smartphone usage patterns.

## Conclusions

Smartphone addiction is prevalent and occurs more frequently amongst younger adults. Proxy measures of screen time were not synonymous with addiction; a validated addiction instrument should be used to capture this phenomenon. Those exhibiting smartphone addiction experienced poorer sleep.

## Data Availability Statement

The raw data supporting the conclusions of this article will be made available by the authors, without undue reservation.

## Ethics Statement

The studies involving human participants were reviewed and approved by Ethical approval was received from the King's College Research Ethics Office (Study ID: 9138; MRS-18/19-9138). Written informed consent for participation was not required for this study in accordance with the national legislation and the institutional requirements.

## Author Contributions

BC, NK, and SS: conceived the study, were responsible first draft of the manuscript, and approved the final draft of the manuscript. BC, NK, LK, and SS: generated the study material. LK and SS: collected the data. BC and SS: analyzed and interpreted the data. BC, NK, PR, and SS: edited the manuscript. BC was the study Guarantor. All authors contributed to the article and approved the submitted version.

## Conflict of Interest

NK was employed by the NHS and was a trustee of the Gordon Moody Association. Her PhD (2010-2013) was joint-funded by the Wellcome Trust and GSK and during that time she received research materials from GSK and educational support from GSK and the Lundbeck Foundation. BC was partially supported by the Biomedical Research Centre at South London and Maudsley NHS Foundational Trust and King's College London. The remaining authors declare that the research was conducted in the absence of any commercial or financial relationships that could be construed as a potential conflict of interest.
