# Supplementary material for: The Association Between Smartphone Addiction and Sleep: A UK Cross-Sectional Study of Young Adults
Source: Front Psychiatry. 2021 Mar 2;12:629407. doi: 10.3389/fpsyt.2021.629407 (PMC7961071; doi:10.3389/fpsyt.2021.629407)
Supplement: Supplementary file 1 [file Table_1.docx]

**Supplementary Table 1: Case Report Form**

**
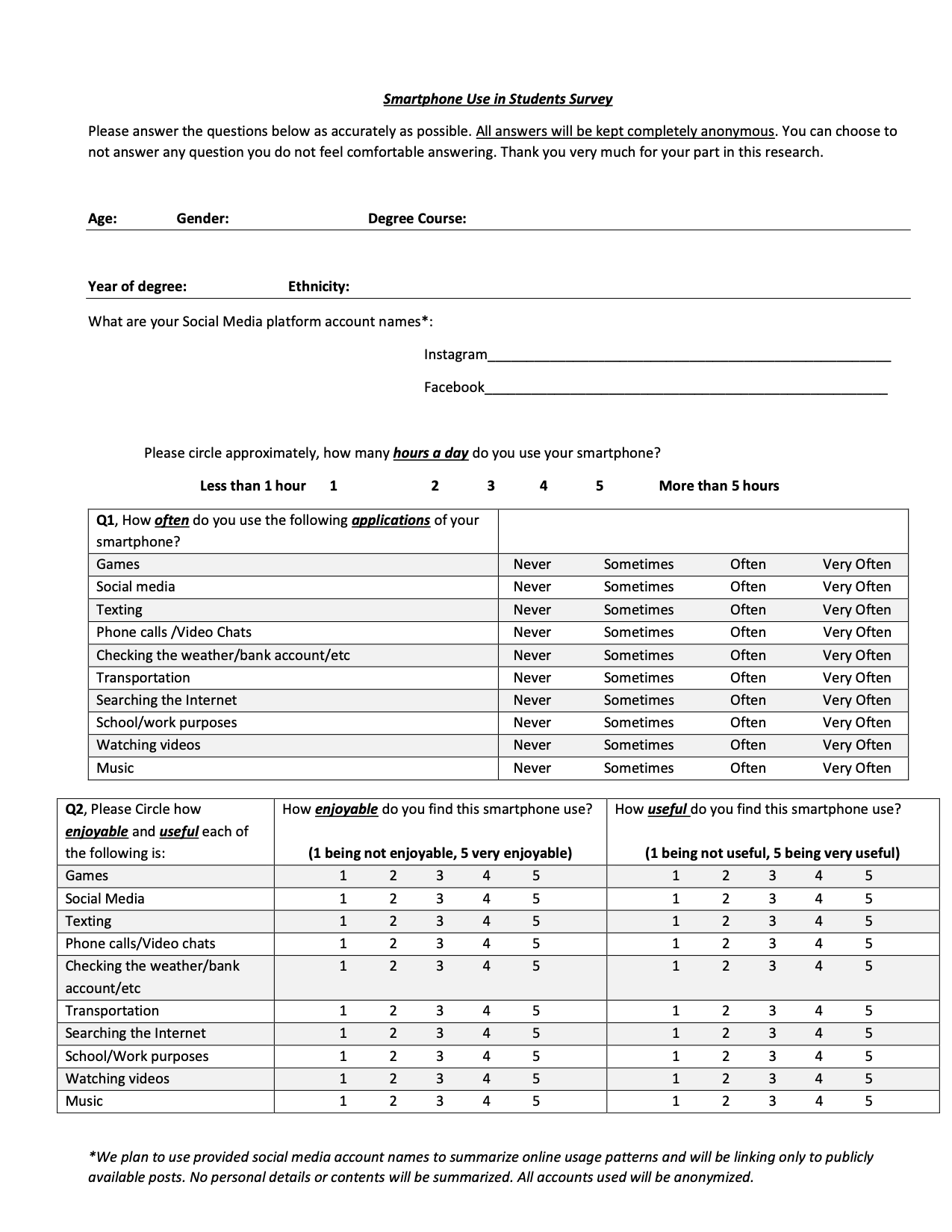
**

**
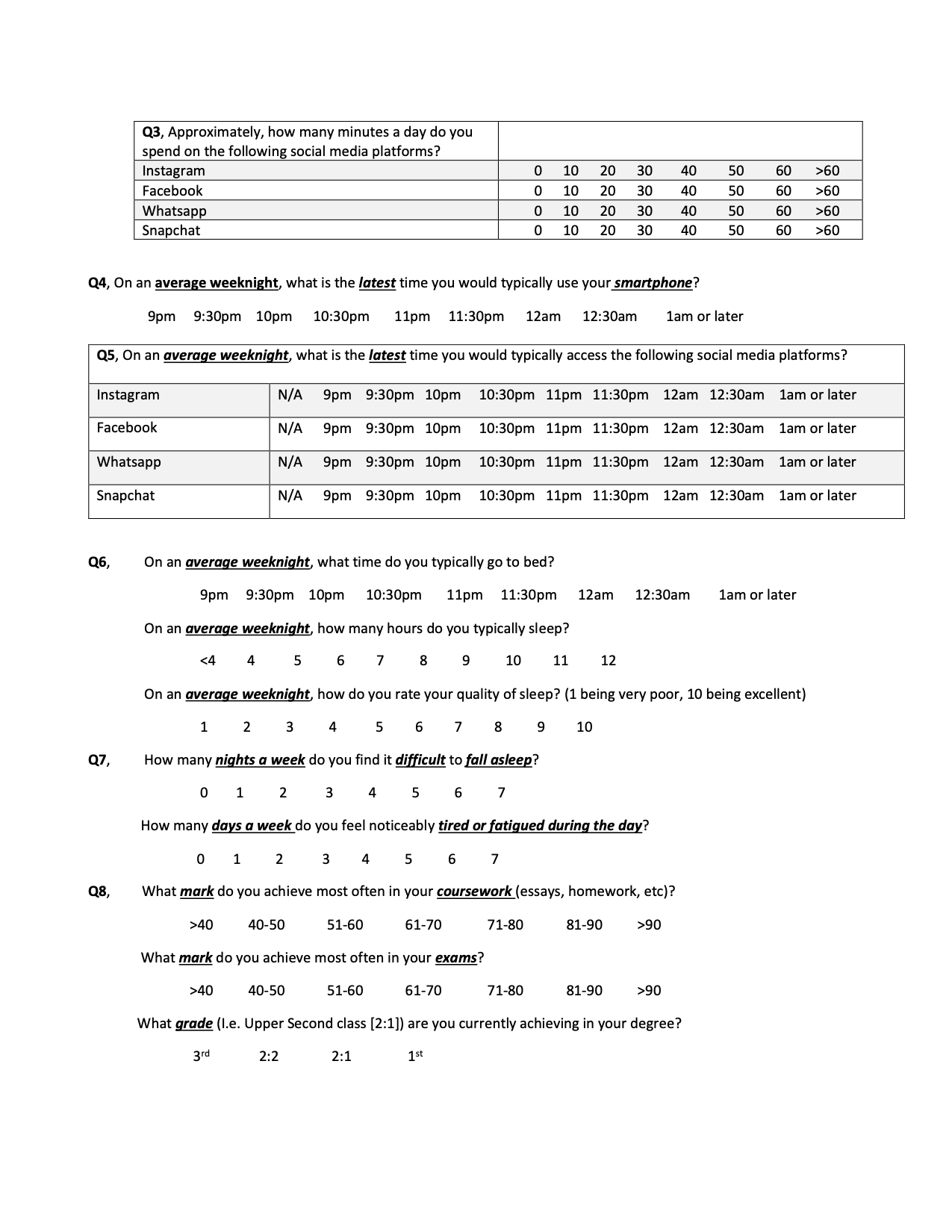

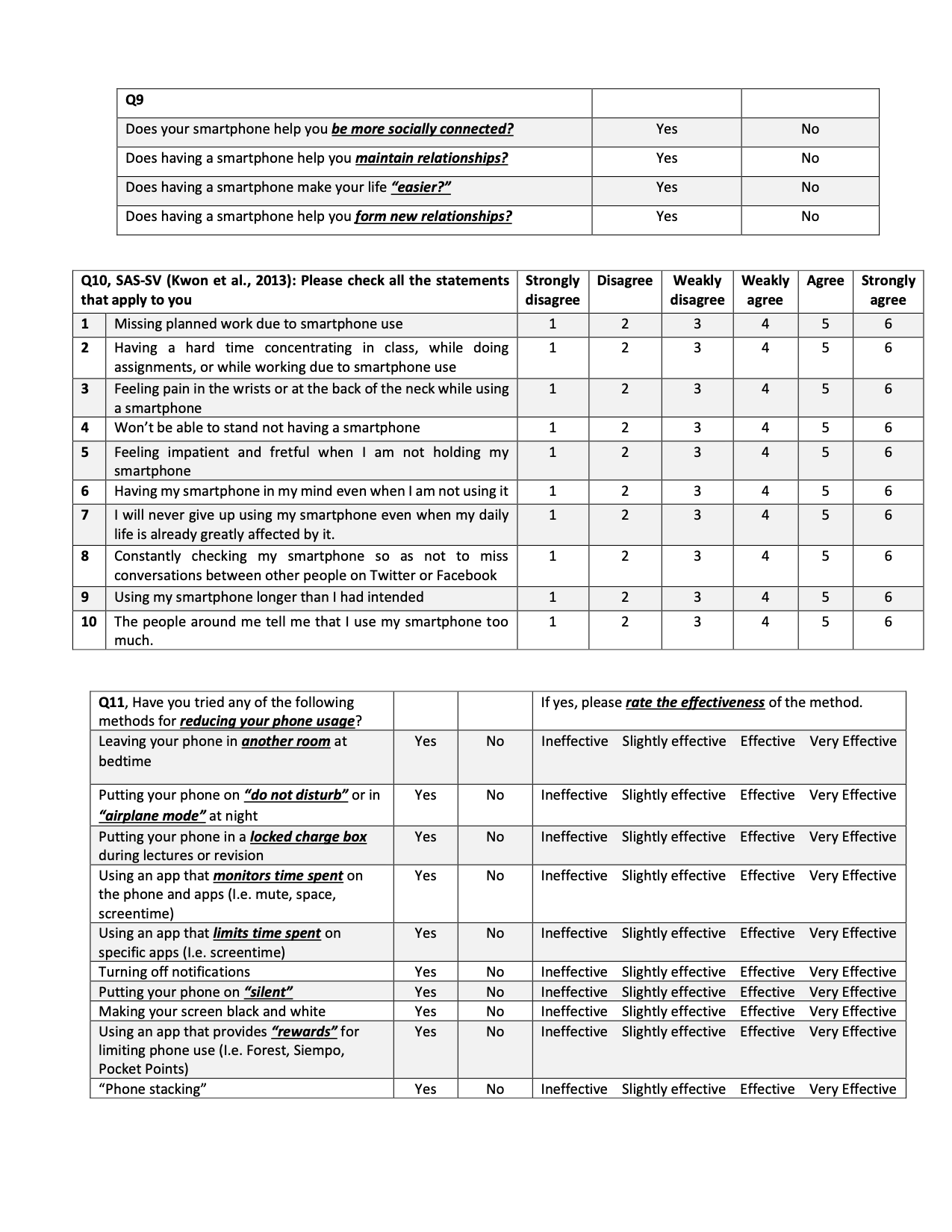
**

**Supplementary Table 2: Reduction strategies used by participants**

| Strategy |  | **Smartphone addiction (SAS-SV)** | | **Sleep score** | |
| --- | --- | --- | --- | --- | --- |
|  | **Participants attempted: n(%)** | **Smartphone Addicted**  **n (%)** | **Not addicted**  **n (%)** | **Good sleep:**  **n (%)** | **Poor sleep:**  **n (%)** |
| A) Leaving your phone in another room at bedtime | 409 (39.2) | 153 (37.4) | 256 (62.6) | 159 (38.9) | 250 (61.1) |
| B) Putting your phone on “do not disturb” or in “airplane mode” at night | 706 (67.7) | 268 (38.0) | 438 (62.0) | 257 (36.4) | 449 (63.6) |
| C) Putting your phone in a locked charge box during lectures or revision | 240 (22.9) | 112 (46.7) | 128 (53.3) | 85 (35.4) | 155 (64.6) |
| D) Using an app that monitors time spent on the phone and apps | 445 (42.7) | 194 (43.6) | 251 (56.4) | 162 (36.4) | 283 (63.6) |
| E) Using an app that limits time spent on specific apps | 266 (25.5) | 117 (44.0) | 149 (56.0) | 87 (32.7) | 179 (67.3) |
| F) Turning off notifications | 713 (68.4) | 278 (39.0) | 435 (61.0) | 267 (37.4) | 446 (62.6) |
| G) Putting your phone on “silent” | 888 (85.1) | 334 (37.6) | 554 (62.4) | 336 (37.8) | 552 (62.2) |
| H) Making your screen black and white | 104 (10.0) | 41 (39.4) | 63 (60.6) | 29 (27.9) | 75 (72.1) |
| I) Using an app that provides “rewards” for limiting phone use | 199 (19.1) | 92 (46.2) | 107 (53.8) | 72 (36.2) | 127 (63.8) |
| J) Phone stacking | 126 (12.1) | 63 (50.0) | 63 (50.0) | 46 (36.5) | 80 (63.5) |

**Supplementary Table 3: Logistic regression analysis of reduction strategies on sleep**^†^ . *p<0.05; **p<0.01.

A=Leaving phone in another room at bedtime

B=Putting phone on do not disturb or airplane mode at night

D=Using an app that monitors time spent on the phone and apps

E=Using an app that limits time spent on specific apps

F=Turning off notifications

G=Putting your phone on silent

H=Making your phone black and white

|  | Sleep (95% CI) | Strategy A | Strategy B | Strategy D | Strategy E | Strategy F | Strategy G | Strategy H |
| --- | --- | --- | --- | --- | --- | --- | --- | --- |
| Age (22-25) | 0.85 (0.64 – 1.13) | 0.86 (0.64 – 1.14) | 0.86 (0.64 – 1.14) | 0.85 (0.64 – 1.13) | 0.85 (0.64 – 1.14) | 0.85 (0.64 – 1.14) | 0.85 (0.64 – 1.13) | 0.85 (0.64 – 1.13) |
| Age ≥ 26 | 0.74 (0.46 – 1.18) | 0.74 (0.47 – 1.19) | 0.74 (0.46 – 1.18) | 0.74 (0.46 – 1.18) | 0.73 (0.46 – 1.16) | 0.74 (0.47 – 1.19) | 0.74 (0.46 – 1.18) | 0.75 (0.47 – 1.19) |
| Females | 1.13 (0.86 – 1.51) | 1.16 (0.87 – 1.54) | 1.12 (0.84 – 1.49) | 1.13 (0.85 – 1.50) | 1.13 (0.85 – 1.51) | 1.14 (0.86 – 1.51) | 1.14 (0.86 – 1.51) | 1.13 (0.85 – 1.50) |
| SAS-SV | 1.61 (1.24 – 2.10)** | 1.58 (1.21 – 2.06)** | 1.61 (1.23 – 2.09)** | 1.60 (1.23 – 2.09)** | 1.59 (1.22 – 2.07)** | 1.60 (1.23 – 2.08)** | 1.61 (1.24 – 2.10)** | 1.60 (1.23 – 2.08)** |
| Strategy A effective |  | 0.64 (0.41 – 0.99)* |  |  |  |  |  |  |
| Strategy B effective |  |  | 0.88 (0.62 – 1.26) |  |  |  |  |  |
| Strategy D effective |  |  |  | 1.04 (0.70 – 1.54) |  |  |  |  |
| Strategy E effective |  |  |  |  | 0.74 (0.44 – 1.24) |  |  |  |
| Strategy F effective |  |  |  |  |  | 0.87 (0.62 – 1.22) |  |  |
| Strategy G effective |  |  |  |  |  |  | 1.03 (0.78 – 1.37) |  |
| Strategy H effective |  |  |  |  |  |  |  | 0.63 (0.28 – 1.45) |

^†^C=Locked charge box; H=Using app that provides rewards for limiting phone use; I=Phone stacking were excluded from this analysis as they were not deemed pertinent to the outcome (sleep).
